# mRNA-1273 and BNT162b2 COVID-19 vaccines elicit antibodies with differences in Fc-mediated effector functions

**DOI:** 10.1126/scitranslmed.abm2311

**Published:** 2022-03-29

**Authors:** Paulina Kaplonek, Deniz Cizmeci, Stephanie Fischinger, Ai-ris Collier, Todd Suscovich, Caitlyn Linde, Thomas Broge, Colin Mann, Fatima Amanat, Diana Dayal, Justin Rhee, Michael de St. Aubin, Eric J. Nilles, Elon R. Musk, Anil S. Menon, Erica Ollmann Saphire, Florian Krammer, Douglas A. Lauffenburger, Dan H. Barouch, Galit Alter

**Affiliations:** ^1^ Ragon Institute of MGH, MIT, and Harvard, Cambridge, MA 02139, USA; ^2^ Center for Virology and Vaccine Research, Beth Israel Deaconess Medical Center, Harvard Medical School, Boston, MA 02215, USA.; ^3^ Seromyx Systems Inc, Cambridge, MA 02139, USA; ^4^ Department of Immunology and Microbial Science, The Scripps Research Institute, La Jolla Institute for Immunology, La Jolla, CA 92037, USA.; ^5^ Department of Microbiology, Icahn School of Medicine at Mount Sinai, New York, NY 10029, USA; ^6^ Space Exploration Technologies Corp, Hawthorne, CA 90250, USA; ^7^ Brigham Women’s Hospital, Boston, MA 02115, USA; ^8^ Department of Biological Engineering, Massachusetts Institute of Technology, Cambridge, MA 02142, USA

## Abstract

The successful development of several coronavirus disease 2019 (COVID-19) vaccines has substantially reduced morbidity and mortality in regions of the world where the vaccines have been deployed. However, in the wake of the emergence of viral variants that are able to evade vaccine-induced neutralizing antibodies, real-world vaccine efficacy has begun to show differences across the two approved mRNA platforms, BNT162b2 and mRNA-1273; these findings suggest that subtle variation in immune responses induced by the BNT162b2 and mRNA-1273 vaccines may confer differential protection. Given our emerging appreciation for the importance of additional antibody functions beyond neutralization, we profiled the post-boost binding and functional capacity of humoral immune responses induced by the BNT162b2 and mRNA-1273 vaccines in a cohort of hospital staff. Both vaccines induced robust humoral immune responses to wild-type severe acute respiratory syndrome coronavirus 2 (SARS-CoV-2) and to variants of concern. However, differences emerged across epitope-specific responses, with higher concentrations of receptor binding domain (RBD)- and N-terminal domain-specific IgA observed in recipients of mRNA-1273. Antibodies eliciting neutrophil phagocytosis and natural killer cell activation were also increased in mRNA-1273 vaccine recipients as compared to BNT162b2 recipients. RBD-specific antibody depletion highlighted the different roles of non-RBD-specific antibody effector functions induced across the mRNA vaccines. These data provide insights into potential differences in protective immunity conferred by these vaccines.

## Teaser

COVID-19 mRNA vaccines induce robust humoral immune responses with differences in epitope recognition and antibody-mediated functional properties.

## INTRODUCTION

The unprecedented and rapid generation of multiple severe acute respiratory syndrome coronavirus 2 (SARS-CoV-2) vaccines marked a breakthrough in vaccine development and provided hope for an end to the coronavirus disease 2019 (COVID-19) pandemic. However, rising numbers of breakthrough infections, driven by evolving variants of concern (VOCs) in the setting of waning immunity, have clearly illustrated the urgent need to define correlates of immunity. Preliminary immune correlates analyses have shown a strong relationship between neutralizing antibody concentrations and vaccine efficacy (*1*). Yet antibody binding titers provide an even stronger surrogate of protection across vaccine platforms (*2*–*4*), with protection observed prior to the evolution of neutralizing antibodies (*4*, *5*). This protection persisted even in the setting of waning neutralizing antibodies (*6*). These data argue for a potential role for alternative protective antibody mechanisms of action.

Beyond their role in binding and neutralization, antibodies mediate a wide array of additional immunological functions through their ability to recruit the immune system using Fc receptors (FcRs) and complement (*7*). Fc-mediated effector functions have been implicated in protection against multiple pathogens, including influenza (*8*), anthrax (*9*), HIV (*10*), malaria (*11*), and Ebola virus (*12*). Likewise, Fc-mediated effector functions have been linked to protection against SARS-CoV-2 both following vaccination and following infection (*13*–*16*). Moreover, these effector functions play a critical role in the therapeutic activity of spike protein-specific monoclonal antibodies, with a more minor, yet defined, role in prophylactic antibody therapy in animal models (*17*). Importantly, Fc-mediated effector functions have been implicated in reducing the severity of disease rather than transmission, and thus may play a more critical role in vaccine attenuated disease, rather than simple blockade of infection. Although accumulating data points to the ability of adenoviral platforms to evoke strong Fc-mediated effector functions (*18*, *19*) that have been linked to protection against HIV or malaria (*20*), less is known about the ability of newer vaccine platforms, including mRNA vaccines, to elicit these functions.

Robust protection was observed in phase 3 trials of Pfizer/BioNTech BNT162b2 (*4*) and Moderna mRNA-1273 (*21*), with 94.1% and 95% vaccine efficacy observed at a time when the D614G strain was dominant in circulation. Yet, despite similar antibody titers and neutralizing antibody concentrations across these mRNA vaccines, emerging real-world effectiveness study have begun to point to differences between vaccines. Specifically, in the face of the Delta variant, about 40% and about 75% efficacy was observed in BNT162b2 and mRNA-1273 vaccinees (*22*, *23*). Preliminary data in pregnant women have also begun to point to differences in vaccine-induced humoral immune responses elicited by BNT162b2 and mRNA-1273 vaccines (*24*); these differences have been proposed to be driven by differences in vaccine dose, formulation, or the one week-delay in boosting (*25*). However, whether similar differences exist in the non-pregnant vaccinees, particularly across VOCs, remains incompletely understood.

Here, we compared the humoral response across the BNT162b2 and mRNA-1273 at peak immunogenicity in a group of hospital workers. Both vaccines induced robust functional humoral immune responses, yet differences were noted in the vaccine-induced antibody profiles across the vaccine groups, with higher receptor binding domain (RBD)- and N-terminal domain (NTD)-specific IgA, as well as functional antibodies, among mRNA-1273 immunized vaccinees. Both mRNA vaccines drove robust responses against VOCs, including the beta and delta variants. Moreover, RBD-specific antibody depletion highlighted the presence of non-RBD-specific antibody effector function deployed by both platforms, albeit at different concentrations, providing evidence to explain the differential Fc-mediated effector functions observed.

## RESULTS

### Study Population

Seventy-three participants were included in this study. Twenty-eight received mRNA-1273 and 45 received BNT162b2. These samples were from a hospital-wide biorepository of vaccinated individuals who received an mRNA COVID-19 vaccine and had serum available for analysis following their second vaccine dose (Table 1). Both vaccines were delivered intramuscularly. Thirty μg of BNT162b2 and 100 μg of mRNA-1273 were delivered three and four weeks apart. Samples were obtained a median (interquartile range, IQR) of 19 (15, 26) days after the second vaccine dose. Prior SARS-CoV-2 infection (mild disease) was diagnosed in 7% of mRNA-1273 vaccinated individuals, and 2% of BNT162b2 vaccinated individuals. After the second dose, fever was reported in 12 (48%) mRNA-1273 vaccinated and 19 (45%) of BNT162b2 vaccinated participants (Table 1).

**Table 1.** Characteristics of vaccinated participants. Participants were chosen from the biorepository based on their sample availability at the time of the analysis. IQR, interquartile range.

	**mRNA-1273 vaccinated** ***n = 28***	**BNT162b2 vaccinated** ***n = 45***
Age, median (IQR), years	32 (26, 42)	32 (27, 37)
Sex at birth, female	23 (82%)	41 (91%)
**Race, No.**	25	42
White	20 (80%)	29 (69%)
Black	0	5 (12%)
Asian Multi-racial Other	2 (8%) 2 (8%) 2 (8%)	3 (7%) 4 (10%) 1 (2%)
**Ethnicity,** No.	26	41
Hispanic or Latino	3 (12%)	6 (15%)
**Chronic medical conditions** Diabetes Hypertension Obesity (body mass index ≥ 30mg/kg^2^) Asthma	0 2 (7%) 3 (11%) 3 (11%)	1 (2%) 3 (7%) 2 (3%) 3 (7%)
Prior SARS-CoV-2 infection	2 (7%)	1 (2%)
Days from second vaccine dose to sample collection, median (IQR)	17 (15-20)	21 (17-27)
**Fever within 48 hours (by self-report)** After first dose, n/N After second dose, n/N	1/24 (4%) 12/25 (48%)	0/41 (0%) 19/41 (46%)

### mRNA-1273 and BNT162b2 COVID-19 vaccines induce robust antibody responses to D614G SARS-CoV-2.

**Fig. 1.**
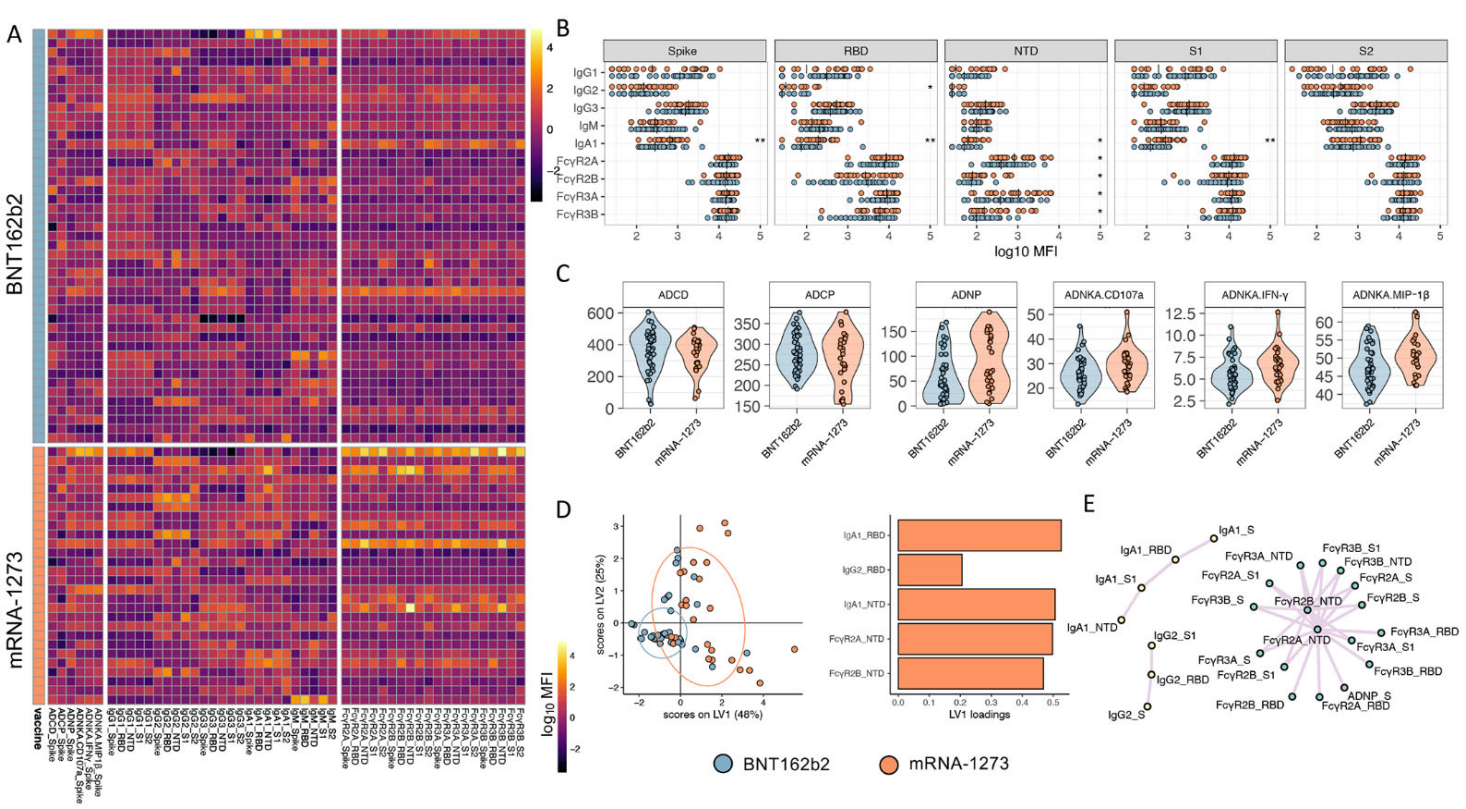
**mRNA-1273 and BNT162b2 COVID-19 vaccines induce similar SARS-CoV-2 D614G-specific antibody profiles. (A)** A heatmap is shown summarizing SARS-CoV-2 D614G spike protein, RBD, NTD, S1, and S2-specific IgG1, IgG2, IgG3, IgA1, and IgM titers, Fc measurements, and functional assays (antibody-dependent complement deposition (ADCD), cellular phagocytosis (ADCP) and neutrophil phagocytosis (ADNP), and NK cell activation (ADNKA, CD107a surface expression, and MIP-1β or IFN-γ production) for each participant in the mRNA-1273 (*n = 28*) and BNT162b2 (*n = 45*) vaccine arms of the study. Titers and FcRs were first log-transformed, and all measurements were z-scored. **(B and C)** Univariate comparisons between BNT162b2 (blue) and mRNA-1273 (red) vaccine recipients are shown for **(B)** antibody titers and FcγR binding for SARS-CoV-2 D614G spike protein-, RBD-, NTD-, S1-, and S2-specific antibodies, as well as **(C)** spike protein-specific antibody-mediated effector functions. Vertical bars in (B) depict median values. Mann-Whitney U-tests corrected for multiple comparisons by the Benjamini-Hochberg (BH) method were used. The adjusted *p* < 0.001 ***, *p* < 0.01 **, *p* < 0.05 *. **(D)** A least Absolute Shrinkage Selection Operator (LASSO) was used to select antibody features that contributed most to the discriminate subjects vaccinated with BNT162b2 or mRNA-1273. A partial least square discriminant analysis (PLSDA) was used to visualize samples. LASSO selected features were ranked based on their Variable of Importance (VIP) score, and the loadings of the latent variable 1 (LV1) were visualized in a bar graph. **(E)** A co-correlate network of the LASSO selected features was built using a threshold of absolute Spearman rho greater than 0.7 and BH-adjusted p-value lower than 0.01. Nodes were colored based on the type of measurement; titers are shown in yellow, FcγRs are shown in green, and functional measurements are shown in gray. All shown links are positive correlations.

The two approved mRNA vaccines are known to induce robust antibody titers and specifically neutralizing antibodies (*26*, *27*); however, real-world efficacy data has begun to show differences across the vaccines in their ability to prevent infection (*22*). Thus, we sought to determine whether the two authorized COVID-19 mRNA vaccines elicited similar Fc profiles. Wild-type (D614G) SARS-CoV-2 RBD-, NTD-, spike protein-, S1-, and S2-specific antibody titers, FcR binding, and Fc effector functions were analyzed. Robust vaccine-induced antibody responses were observed across both the mRNA-1273 (*n = 28*) and BNT162b2 (*n = 45*) vaccines (Fig. 1A, data file S1). Univariate comparisons across each antigen and Fc-profile measurement highlighted the presence of equivalent IgG and IgM binding titers, but significantly higher concentrations of IgA-binding titers elicited by the mRNA-1273 vaccine, particularly to the spike protein (p = 0.008), RBD (p = 0.001), NTD (p = 0.016), and S1 domain (p = 0.003) (Fig. 1B and data file S2). Moreover, robust, and largely equivalent cross FcR binding was observed across both vaccines, with the exception of enhanced NTD-specific Fcγ receptor (FcγR) binding antibodies induced by the mRNA-1273 vaccine (p = 0.016, 0.018, 0.041, and 0.025 for FcγR2A, FcγR2B, FcγR3A and FcγR3B, respectively). Similarly, equivalent degrees of antibody dependent complement deposition (ADCD) and antibody dependent cellular phagocytosis by monocytes (ADCP) were observed across the two vaccine groups at peak immunogenicity. Conversely, mRNA-1273 vaccinated individuals exhibited increased antibody dependent neutrophil phagocytosis (ADNP) activity and antibody dependent natural killer (NK) cell activation (ADNKA) measured by degranulation (CD107a expression), interferon (IFN)-γ secretion, and macrophage inflammatory protein 1β (MIP1-β) secretion (p = 0.051, 0.14,1 0.118 and 0.111 for ADNP and ADNKA CD107a, IFNγ, and MIP1β respectively) (Fig. 1C and data file S2).

Vaccine-specific signatures were further identified using multivariate analysis. Multivariate approaches evaluate the classification accuracy of samples based on multiple features simultaneously, providing a deeper understanding of the compound differences in antibody responses that go beyond simple univariate differences. Specifically, unsupervised Principal Component Analysis (PCA) (fig. S1) and supervised least absolute shrinkage and selection operator (LASSO) feature selection coupled to visualization with partial least squares discriminant analysis (PLSDA) provides multivariate resolution to determine whether overall antibody profiles differ across the vaccines (Fig. 1D). Interestingly, a partial separation between the mRNA vaccines was observed using an unsupervised PCA. mRNA vaccine profiles separated along the Principal Component 1 (PC1), with an accumulation of BNT162b2 vaccinated antibody profiles in the bottom right side of the PCA plot (fig. S1). Additionally, LASSO-PLSDA analysis identified two distinct mRNA vaccine profiles (Fig. 1D), marked largely by augmented responses in the mRNA-1273 vaccine-induced immune response. Specifically, five features were selectively enhanced in the mRNA-1273 vaccine profiles, including RBD-specific IgA1 and IgG2, as well as NTD-specific IgA1, FcγR2A and FcγR2B binding. Given the correlated nature within the vaccine-induced humoral immune responses, a correlation network analysis was built between LASSO-selected features and the overall immune response to further define any additional antibody features that shifted selectively across the vaccine profiles (Fig. 1E). Three clusters appeared: a cluster enriched for elevated IgA responses across all antigenic determinants, a smaller network of IgG2 responses, and a large network of FcR-binding antibody responses across multiple antigenic targets all enriched among mRNA-1273 immunized individuals. These data point to robust humoral immune responses induced by both mRNA platforms, with potentially enhanced NTD recognition, IgA immunity, and specific antibody effector functions enriched among mRNA-1273 immunized individuals compared to BNT162b2 recipients.

### mRNA-1273 or BNT162b2 vaccination induces FcR-binding responses to multiple VOCs.

**Fig. 2.**
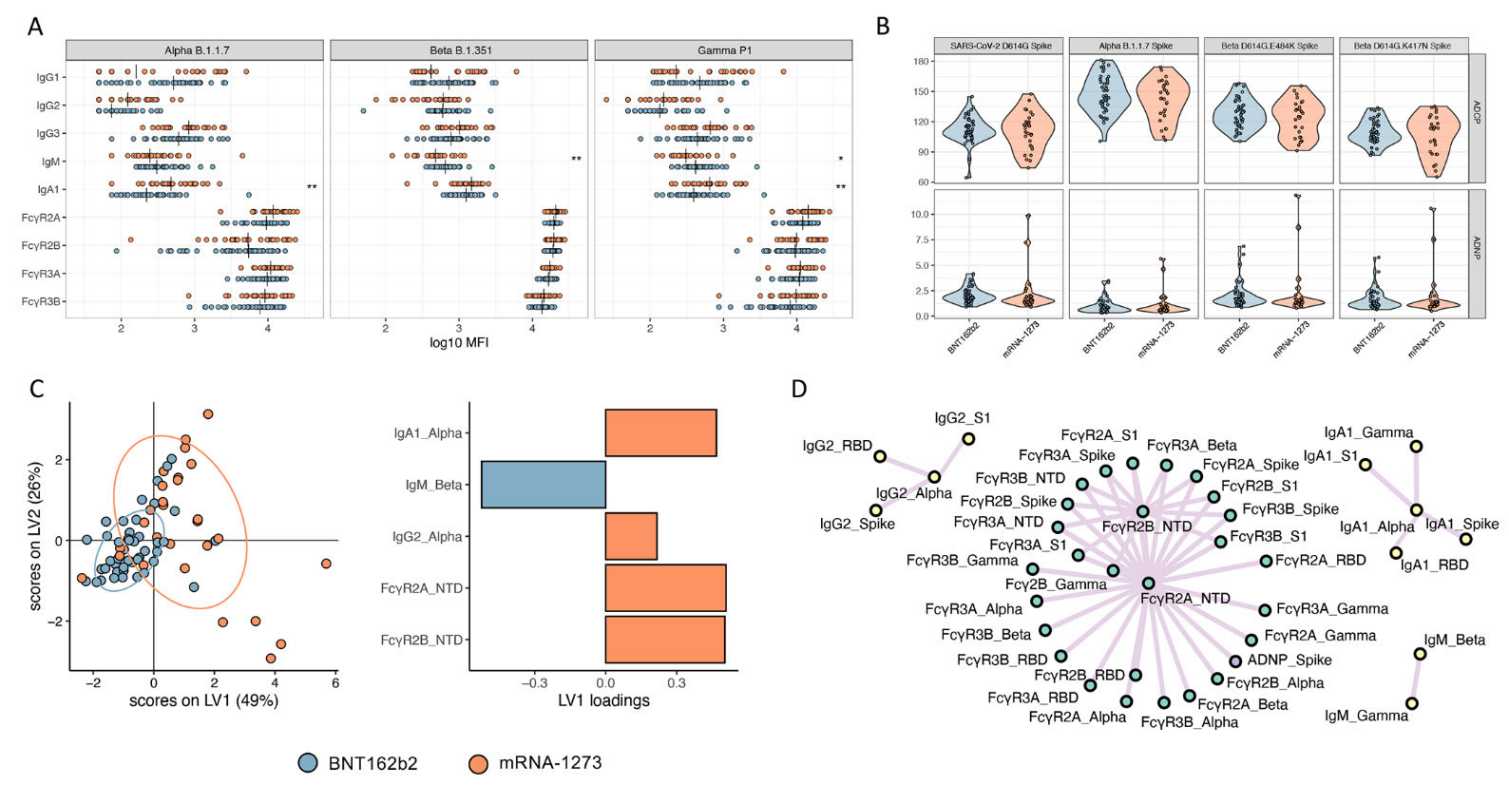
**mRNA-1273 and BNT162b2 vaccines induce a comparable antibody profile across Alpha, Beta, and Gamma SARS-CoV-2 VOCs. (A and B)** Univariate comparisons of **(A)** antibody titers and FcγR binding for SARS-CoV-2 Alpha, Beta, and Gamma VOCs as well as **(B)** ADCP and ADNP activity are shown for BNT162b2 (blue) and mRNA-1273 (red) vaccinated individuals. Vertical bars in (A) depict median values. Mann-Whitney U-tests corrected for multiple comparisons with the Benjamini-Hochberg (BH) method were used. Adjusted p-values are indicated as ***p < 0.001, **p < 0.01, and *p < 0.05. **(C)** A LASSO-PLSDA model is shown including all variant measurements. LASSO selected features were ranked based on their Variable of Importance (VIP) score, and the loadings of the latent variable 1 (LV1) were visualized in a bar graph. **(D)** A co-correlate network of the LASSO-selected features was built using a threshold of absolute Spearman rho greater than 0.7 and BH-adjusted p-value lower than 0.01. Nodes were colored based on the type of measurement; titers are shown in yellow, FcγRs are shown in green, and functional measurements are shown in gray. All shown links are positive correlations.

Despite the remarkable efficacy of the mRNA vaccines against SARS-CoV-2 D614G, waves of variants have emerged that include amino acid substitutions that diminish neutralizing antibody activity (*28*–*30*). Among the VOCs, the mRNA vaccines appear to neutralize Alpha (B.1.1.7) (*31*) and Gamma (P.1) with only a minimal loss of activity, but exhibit compromised neutralizing activity against the beta (B.1.351) variant (*32*, *33*). Yet, whether Fc responses were equally affected across the VOCs remains unclear. Both mRNA-1273 and BNT162b2 vaccine-induced antibodies bound equally well across the Alpha, Beta, and Gamma VOCs (Fig. 2A). Interestingly, Beta- and Gamma-specific IgM titers were higher in BNT162b2 vaccinated individuals (p *=* 0.008 and 0.022, respectively). Conversely, IgA responses were amplified in the mRNA-1273 immunized individuals for Alpha and Gamma VOCs (p *=* 0.001 and 0.008, respectively) (Fig. 2A and data file S2). However, FcR-binding antibodies targeting all three VOCs were comparably induced by both vaccines. Similarly, both antibody-dependent monocyte (ADCP) and neutrophil (ADNP) phagocytosis were largely equivalent across the variants (Fig. 2B and data file S2), highlighting the robust FcR-binding and functional profiles across VOCs elicited by both mRNA platforms. We next asked if any multivariate differences could be observed across the two mRNA platforms in terms of responses to VOCs (Fig. 2C). LASSO-PLSDA revealed separation in the Fc profiles between the mRNA-1273 and BNT162b vaccinated individuals in response to VOCs (Fig. 2C). The profile was marked by increased IgM responses to the Beta variant in BNT162b vaccinated individuals. Conversely, higher Alpha variant-specific IgA and IgG2 responses were observed in mRNA-1273 vaccinated individuals; these individuals also had a higher abundance of FcγR2A- and FcγR2B-binding antibodies that recognized the NTD. The extended LASSO co-correlate network further highlighted the presence of IgG2-, IgA-, and IgM-only networks across multiple VOCs, suggesting that isotype-biased selection across the mRNA platform includes reactivities across VOCs (Fig. 2D). Additionally, a large network of highly functional pan-VOC and epitope responses were observed in the mRNA-1273 profile, marked by an enrichment of NTD-specific antibody responses. Thus, the two mRNA platforms elicit an overall similar abundance of functional antibodies to the VOCs, with an IgM- and IgG-biased profile induced by BNT162b2 vaccination and a more class-switched IgA- and IgG-driven profile induced by mRNA-1273 vaccination.

### mRNA-1273 and BNT162b2 vaccines induce more robust Fc-functional antibodies compared to infection.

**Fig. 3.**
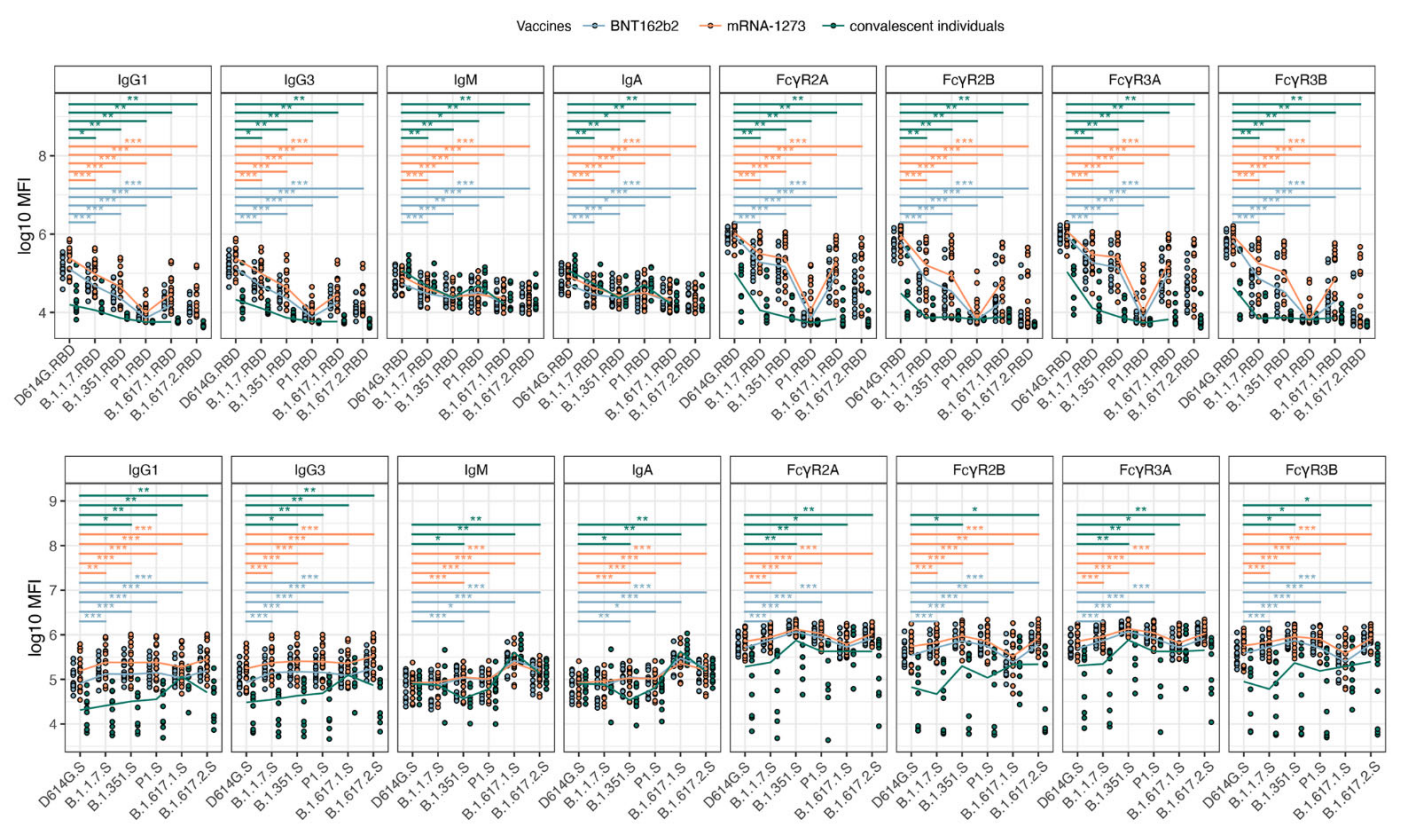
mRNA vaccines are able to induce high spike protein-specific antibody binding to FcRs across all SARS-CoV-2 VOCs. Univariate comparisons between BNT162b2 (blue) vaccine recipients, mRNA-1273 (red) vaccine recipients, and convalescent individuals (green) are shown for RBD-specific (top row) and full-length spike (S) protein-specific (bottom row) IgG1, IgG2, IgG3, IgA1, and IgM titers as well as FcγR binding. Comparisons are shown for SARS-CoV-2 D614G and indicated VOCs. Lines connect median values for each cohort. Comparisons were made for each variant using Wilcoxon rank-sum tests and corrected for multiple comparisons using the Benjamini Hochberg (BH) method. T Adjusted p-values are indicated as ***p < 0.001, **p < 0.01, and *p < 0.05.

Several reported VOCs have been reported to break through both infection-elicited (*34*) and vaccine-induced immune responses (*30*), causing large numbers of outbreaks. Evidence showing the reduction of effectiveness of mRNA vaccines against VOCs, especially the Delta variant, is emerging (*22*), albeit with the majority of breakthroughs remaining largely non-lethal (*1*). Yet, differential real-world efficacy against the Delta VOC (*22*) points to a nuanced immune response to Delta. Thus, we next aimed to compare the cross-VOC antibody Fc-profiles targeting both VOC RBDs or full-length spike protein antigens across a subset of the vaccinees and using a group of individuals with a prior case of mild, community-acquired SARS-CoV-2 infection. Antibody profiles were compared across mRNA-1273 (*n = 16*) and BNT162b (*n = 15*) vaccines and 10 convalescent individuals (Fig. 3 and data file S3). mRNA vaccine-induced IgG1 and IgG3 responses were higher than infection-induced responses for the D614G RBD (p = 0.00009 and p = 0.0004 for mRNA-1273 and BNT162b, respectively), and all VOCS: Alpha, Beta, Gamma, Kappa, and Delta RBD (data file S3) and bound to all VOC RBDs less than to D614G (Fig. 3 and data file S3). Similar patterns were observed across all RBD-specific FcR-binding antibodies induced by the mRNA vaccines; VOC-reactive vaccination-elicited antibodies showed superior binding to FcRs as compared to infection-elicited antibodies, which also bound poorly to all VOC RBDs as compared to the D614G virus (Fig. 3, top row, and data file S3). Slightly higher antibody binding was noted across VOC RBDs for samples from mRNA-1273-immunized individuals than BNT162b2, though the recognition pattern was the same (data file S3). Conversely, IgG1 and IgG3 spike protein-specific antibodies exhibited enhanced mRNA binding to nearly all full-length spike protein antigens from VOCs, except the Kappa variant, as compared to D614G spike protein (Fig. 3, bottom row, and data file S3). Importantly, all spike protein-specific binding IgG responses were lower in convalescent individuals compared to mRNA vaccinees (data file S3). Thus, despite the more variable FcR binding profiles specific to VOC RBDs, stable FcR binding was observed for most full-length spike proteins. Given the persistent protection against Delta in recently vaccinated individuals (*1*), but enhanced chance of breakthrough infection over time, these data may suggest that the presence of non-RBD-specific antibodies may be key to durable protection.

### RBD-specific antibody depletion influences antibody-mediated effector function.

**Fig. 4.**
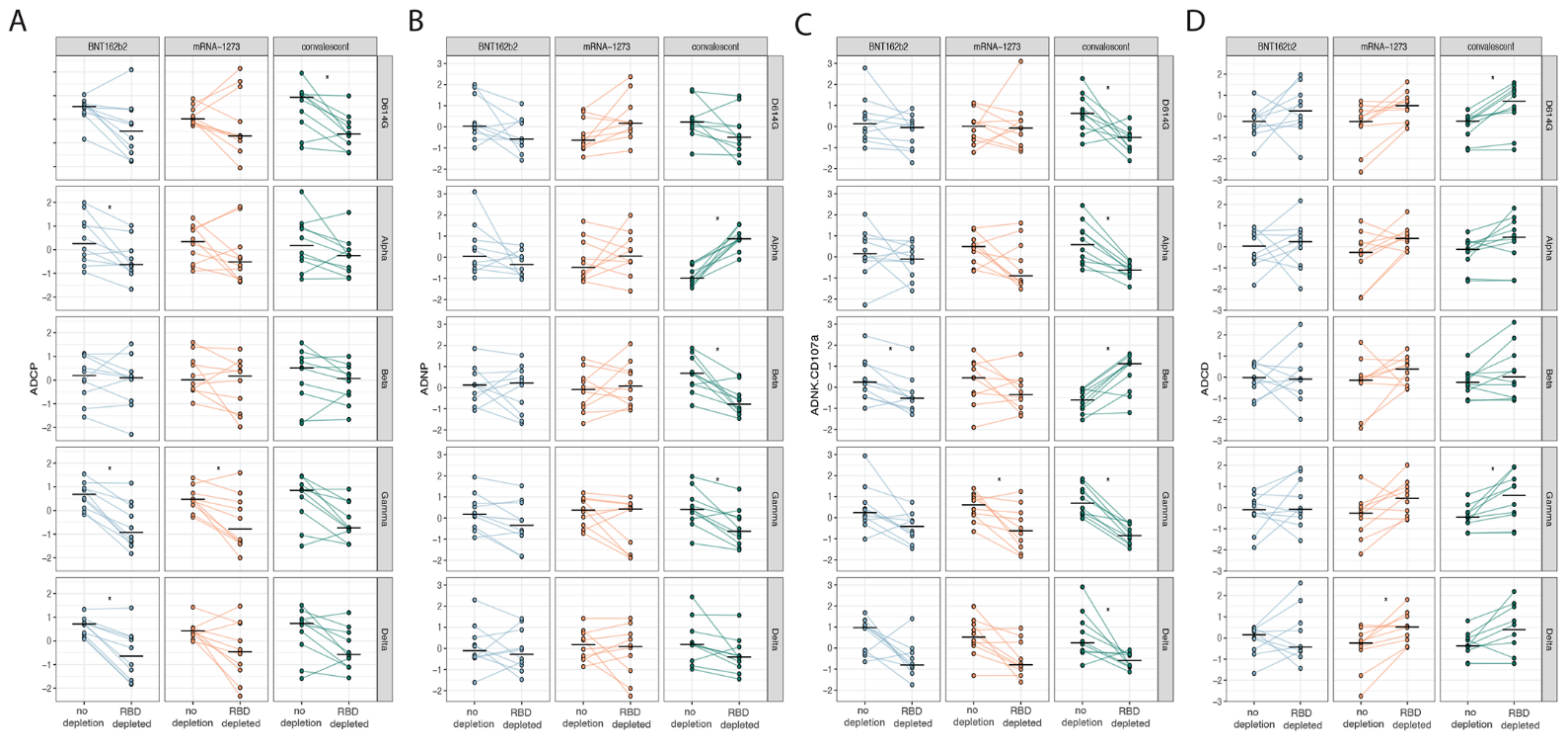
**RBD-specific antibody depletion influences antibody-mediated monocyte and neutrophil phagocytosis following vaccination and infection. (A)** Antibody-mediated cellular phagocytosis (ADCP), **(B)** antibody-mediated neutrophil phagocytosis (ADNP), **(C)** antibody-mediated NK activation (ADNKA) and **(D)** antibody-mediated complement deposition (ADCD), activity specific to the spike protein were compared across SARS-CoV-2 D614G and Alpha, Beta, Gamma, and Delta variants. Comparisons were made between pre-depletion (RBD+) and post-RBD-specific antibody depletion (RBD-) serum samples from BNT162b2 (blue) and mRNA-1273 (red) vaccine recipients and convalescent individuals (green). All data were Z-scored. A paired *t* test, corrected for multiple comparisons by the Benjamini-Hochberg (BH) method, was used to identify differences in non-depleted and depleted plasma. Adjusted p-values are indicated as ***p < 0.001, **p < 0.01, and *p < 0.05.

The data above suggested that potential differences in RBD and spike-protein specific contributions to polyclonal antibody Fc-binding profiles and function. Thus, to address this possibility, RBD-specific antibodies were depleted from polyclonal serum from our vaccinees and convalescent samples (fig. S2) and tested for antibody-mediated effector functions (*13*, *14*). Specifically, ADCP (Fig. 4A), ADNP (Fig. 4B), ADNKA (Fig. 4C), and ADCD (Fig. 4D) activity of non-RBD-specific antibodies were evaluated using spike proteins from the Alpha, Beta, Gamma, and Delta VOCs. RBD-specific antibody depletion resulted in a loss of ADCP activity against D614G, Alpha, Gamma, and Delta variant spike proteins (Fig. 4A) for BNT162b2 vaccinees and convalescent individuals, with a more heterogeneous loss of ADCP in mRNA-1273 immunized individuals. Depletion of RBD-specific antibodies did not reduce ADCP activity against the Beta VOC. In contrast to ADCP, more variation was observed in neutrophil phagocytic activity (ADNP) after RBD-specific antibody depletion particularly in the cohort of convalescent individuals across the Alpha, Beta and Gamma VOCs (Fig. 4B). ADNKA displayed more heterogeneous effect of RBD-specific antibody depletion on ADNKA across VOCs (Fig. 4C). Specifically, samples from BNT162b2 vaccinated individuals exhibited a decrease in ADNKA activity in response to Beta spike protein; samples from recipients of mRNA-1273 exhibited reduced ADNKA activity in response to Gamma variant spike protein. Convalescent plasma depleted of RBD-specific antibodies lost ADNKA activity against D614G, Alpha, Gamma, and Delta variant spike proteins, but showed increased activity against the Beta variant spike protein (Fig. 4C). ADCD exhibited a more uniform responses; RBD-specific antibody depletion in some cases enhanced ADCD (Fig. 4D). Specifically, ADCD activity in response to the Delta variant spike protein was augmented for mRNA-1273 plasma samples, and D614G and gamma variant-specific ADCD activity was increased for convalescent plasma after RBD-specific antibody depletion. These data highlight differences in the functional contribution of RBD-specific antibodies to polyclonal SARS-CoV-2 antibody functionality across the mRNA platforms or after infection. These data may help explain differences in response to VOCs that largely accumulate mutations in the RBD. Together, these data show the robust induction of distinct, functional antibody responses following mRNA-1273 or BNT162b2 vaccination.

## DISCUSSION

Despite the remarkable protective immunity observed in BNT162b2 and mRNA-1273 vaccine studies against the original SARS-CoV-2 variant, breakthrough infections are on the rise globally among vaccinees (*35*). However, severe disease, hospitalization, and death remain low in most populations, except older populations, prompting discussions on additional vaccine boosters (*36*). Although the BNT162b2 and mRNA-1273 vaccines induced comparable total and neutralizing antibody, emerging data point to variation in real-world vaccine effectiveness across the platforms (*22*, *23*, *37*). These differences could be linked to differences in the formulation, design, boosting intervals, and dose, among other features. Thus, understanding immunological differences between these vaccines may provide critical insights on immune correlates of protection; these correlates may then guide next generation vaccine design and further boosting strategies. Among the proposed non-neutralizing antibody immune mechanisms of protection, T cells have been proposed as a critical arm in the control of SARS-CoV-2 infection (*38*, *39*). Yet, additional mechanisms, such as the role of antibody-mediated effector function, have also been shown to play a critical role in vaccine-mediated protection against SARS-CoV-2 (*40*). Thus, we probed the functional humoral immune response induced by distinct mRNA vaccine platforms and measured their Fc-functional performance across spike proteins and RBDs from VOCs. These results demonstrate robust, but distinct, Fc-functional responses induced by the vaccines and point to a potential role for Fc-mediated effector functions in mRNA vaccine-induced protection against disease mediated by VOCs. Further, differences in functional responses to VOCs may contribute to differences in real-world effectiveness across the platforms.

Although no difference in neutralizing activity has been reported across the BNT162b2 and mRNA-1273 vaccines (*27*), differences were observed across the two platforms in this study, both in terms of isotype or subclass and in terms of Fc functions. Whether these differences account for biological variation in real-world protection remains unclear but have been implicated in variation in upper and lower respiratory tract control of SARS-CoV-2 in animal models of vaccine protection (*15*, *41*, *42*). Consistent with previous observations in pregnant women (*16*), elevated concentrations of IgA were noted following mRNA-1273, accompanied by higher ADNP and ADNKA activity. Additionally, it has been shown that IgA possesses great antiviral properties for SARS-CoV-2, and IgA responses may be particularly valuable for vaccine efficacy (*43*–*45*). IgA may contribute to Fc-mediated effector functions through the activation of the high affinity Fcα receptors found on neutrophils. Moreover, changes in both IgA and FcγR activity could collectively shape differences in vaccine response to the virus over time, particularly in light of VOCs that are able to evade neutralizing antibody activity. Conversely, an IgM and IgG bias was noted in samples from BNT162b2 vaccinated individuals in response to VOCs, pointing to differences in class switching across the mRNA platforms. Whether these differences contribute to different degrees of protection, particularly over time as the response wanes, remains unclear but will be addressed in long-term follow-up breakthrough studies. Moreover, whether these differences are related to differences in lipid nanoparticle composition, mRNA dose, or delays in boosting remains unclear, but highlights the potential of mRNA vaccines to drive “tunable” Fc effector functions that may be shaped to achieve selective responses to particular target pathogens and non-infectious diseases in the future.

The majority of mutations in the VOCs occur in NTD and RBD (*46*), which play a critical role in enhancing binding to angiotensin converting enzyme 2 (ACE2). Given that neutralizing antibodies target these same sites, aimed at occluding ACE2 access or at compromising RBD structure, these mutations may compromise neutralizing antibody activity. Conversely, Fc-functional antibodies can target the whole surface of the spike antigen; they are thus not compromised in the same manner as neutralizing antibodies by mutations found to alter ACE2 binding. In fact, although a large fraction of neutralizing antibodies targets the RBD, RBD-specific antibody depletion did not have a major effect on Fc-mediated effector function in BNT162b2 and mRNA-1273 vaccines recipients. Vaccine-induced RBD-specific antibodies influenced ADCP activity following BNT162b2 vaccination, but not other functions. mRNA-1273 induced responses were minimally impacted by RBD depletion. Conversely, convalescent plasma samples were affected by RBD-specific antibody depletion. These data highlight different RBD-centric humoral immune responses across mRNA vaccines and infection, potentially highlighting the more robust protection against VOCs observed following mRNA vaccination as compared to individuals who have infection-elicited immunity. Whether these data point to broader epitope-specific programming by mRNA vaccination compared to infection or the generation of more flexible antibodies remains to be determined. Interestingly, the improved ADCD activity observed following depletion of RBD-specific antibodies also highlighted the possibility that particular antibody subpopulations may block Fc-mediated effector functions, either by blocking access of additional functional antibodies to their epitope or due to changes in Fc-domain of antibodies specific to immunodominant sites, like the RBD, following vaccination or infection. Thus, these data point to differences in Fc programming induced by vaccination and infection in an epitope-specific manner, which may play a critical part in the degree of protection against disease conferred by prior infection or vaccination. Given that neutralizing antibodies depend on the RBD recognition, these data suggest that even with a reduction in the RBD-specific antibody abundance, mRNA vaccine-induced antibodies will continue to elicit robust Fc-mediated effector functions against neutralizing antibody-resistant VOCs, such as Omicron. Although these remaining non-neutralizing mRNA vaccine-induced antibodies will not block transmission, they could still confer protection through rapid clearance of the virus.

The rapid spread of the Delta and Omicron VOCs has raised concerns globally about vaccine efficacy and the need for additional boosters. However, in previously vaccinated individuals, most Omicron infections do not require hospitalization (*47*–*49*). Whether this relates to the less pathological profile of Omicron or to persistent protection afforded by vaccines in the absence of neutralization remains unclear. Although most infection-acquired and vaccine-induced antibodies have lost the ability to prevent transmission against Omicron, vaccine-induced antibodies may still directly contribute to disease attenuation through Fc-mediated effector functions that may clear infection after transmission. Thus, understanding the differences in disease attenuating, and not simply blocking antibodies, elicited by BNT162b2 and mRNA-1273 may provide new clues for the redesign of vaccines and monoclonal therapeutics able to offer a durable barrier of protection against the virus. Linked to our emerging appreciation for the role of Fc-mediated effector functions in protection from disease in nonhuman primates (*42*, *50*), hamsters (*51*), and mice (*17*), upcoming immune correlates analyses and breakthrough studies will provide additional insights into the role of Fc-mediated effector function in protection against SARS-CoV-2.

There are several limitations to our study. First, the mRNA vaccinated individuals were part of a hospital-wide vaccination clinic and were mostly healthy young female health care workers (median age 32 for both mRNA-1273 and BNT162b2 group) from a single site. This prevented the analysis of demographic, age, or co-morbidity related differences in vaccine responses. However, previous studies suggest that age and sex minimally influence the mRNA vaccine response (*52*). Moreover, whether these response differences are durable was not addressed in this analysis but may be critical to further understand the durability of protection over time. Finally, whether these differences translate to real-world efficacy differences across the vaccine platforms remains unclear. Future analysis of immune correlates and real-world effectiveness studies will provide essential insights into the specific differences or common mechanisms that may be key to providing protection from infection or disease. Finally, it is unclear whether differences in the vaccine dose, interval of vaccination between the two doses, mRNA modifications, or lipid nanoparticle formulation drive the observed differences in the vaccine profiles. Yet, despite these limitations, these data provide evidence for potential nuanced differences in the quality of the humoral immune response induced by SARS-CoV-2 mRNA vaccines.

In conclusion, both mRNA vaccines induced robust functional humoral immune responses, with differences in epitope recognition and antibody-mediated functional properties. Additionally, strong responses against VOCs, including the beta and delta variants, were observed. RBD-specific antibody depletion highlighted the different roles of non-RBD-specific antibody effector functions induced across the mRNA vaccines, which might provide insights into potential differences in protective immunity conferred by these vaccines.

## MATERIALS AND METHODS

### Study Design

The Beth Israel Deaconess Medical Center institutional review board approved this study (#2021P000344) and the parent biorepository study (#2020P000361); participants provided written informed consent. Hospital staff of at least 18 years of age planning to receive an mRNA COVID-19 vaccine from December 2020 through February 2021 were enrolled and samples were collected in a more extensive hospital-wide, prospective data and tissue biorepository. Participants self-referred from flyers posted in the hospital vaccine clinics. All participants provided blood samples collected close to each vaccine dose and two to eight weeks after the second dose for the mRNA-1273 or BNT162b2 vaccine. Seventy-three participants, 28 receiving mRNA-1273 and 45 receiving BNT162b2, were included here, providing over 90% power to detect significant differences, based on a 10,000 Monte Carlo simulation and two-sided Wilcoxon rank sum test with type 1 error rate of 5% using previously acquired data (*16*). The analysis presented here includes non-pregnant individuals without immunosuppression medication use. To further characterize the study population, participants were asked to provide their race and ethnicity-based on specified categories for each; they could select multiple race categories. Participants also reported if they had fever symptoms following either vaccine dose.

Industry employees (Space Exploration Technologies Corp.) were volunteer-tested for COVID-19, starting in April 2020. Participants completed a study survey including the collection of COVID-19-related symptoms. Blood samples were collected for serology quarterly. The cohort largely included mild, symptomatic infections (*53*). Although the precise date of infection was not known, routine testing, every three months, placed infections at no more than three months from the sampling date, at a time when humoral immune responses were relatively stable (*53*–*55*). The median age of the seropositive population was 32 years (range 19 to 62 years), and 84% were males. The enrolled participants were 66% White, 8% Asian, 6% more than one race, 2% Black, 1% American Indian/Alaska Native, and 17% unknown. Volunteers were tested by polymerase chain reaction (PCR) and for antibodies monthly. All antibody-positive individuals were included in the study (*53*). Both studies were randomized, blinded prior to final analysis, and approved by the Massachusetts General Brigham Healthcare (previously Partners Healthcare) Institutional Review Board. All participants provided written informed consent.

### Antigens

Antigens used for Luminex based assays included SARS-CoV-2 D614G spike protein (kindly provided by Erica Saphire, La Jolla Institute for Immunology), SARS-CoV-2 S1 protein (Sino Biological), SARS-CoV-2 S2 protein (Sino Biological), and SARS-CoV-2 RBD (kindly provided by Aaron Schmidt, Ragon Institute), as well as antigens from SARS-CoV-2 VOCs, such as Alpha (B.1.1.7) spike protein (LakePharma), Beta (B.1.351) spike protein (LakePharma), Gamma P1 spike protein (LakePharma), Kappa B.1.617.1 spike protein (Sino Biological) and Delta B.1.617.2 spike protein (kindly provided by Erica Saphire, La Jolla Institute for Immunology). Alpha (B.1.1.7), Beta (B.1.351), Gamma P1, Kappa (B.1.617.1) and Delta (B.1.617.2) RBDs were kindly provided by Florian Krammer, Icahn School of Medicine at Mount Sinai.

### Luminex profiling

Serum samples were analyzed by customized Luminex assay to quantify the relative concentration of antigen-specific antibody isotypes, subclasses, and FcγR binding profiles, as previously described (*56*, *57*). Briefly, SARS-CoV-2 antigens were used to profile specific humoral immune responses. Antigens were coupled to magnetic Luminex beads (Luminex Corp) by carbodiimide- N-Hydroxysuccinimide (NHS) ester-coupling (Thermo Fisher Scientific). Antigen-coupled beads were washed and incubated with plasma samples at an appropriate sample dilution (1:500 for IgG1 and all low-affinity FcγRs, and 1:100 for all other readouts) for 2 hours at 37°C in 384-well plates (Greiner Bio-One). The high-affinity FcγR was not tested due to its minimal role in tuning antibody effector function (*58*). Unbound antibodies were washed away, and antigen-bound antibodies were detected using a phycoerythrin (PE)-coupled detection antibody for each subclass and isotype (IgG1, IgG3, IgA1, and IgM; Southern Biotech); FcγRs were fluorescently labeled with PE before addition to immune complexes (FcγR2a, FcγR3a; Duke Protein Production facility). After one hour of incubation, plates were washed, and flow cytometry was performed with an iQue (Intellicyt). Analysis was performed on IntelliCyt ForeCyt (v8.1). PE median fluorescent intensity (MFI) is reported as a readout for antigen-specific antibody titers.

### Analysis of ADCD

Antibody-dependent complement deposition (ADCD) was conducted as previously described (*59*). Briefly, SARS-CoV-2 antigens were coupled to magnetic Luminex beads (Luminex Corp) by carbodiimide-NHS ester-coupling (Thermo Fisher Scientific). Coupled beads were incubated for 2 hours at 37°C with serum samples (1:10 dilution) to form immune complexes and then washed to remove unbound immunoglobulins. In order to measure antibody-dependent deposition of complement component 3 (C3), lyophilized guinea pig complement (Cedarlane) was diluted in gelatin veronal buffer with calcium and magnesium (GBV++) (Boston BioProducts) and added to immune complexes. Subsequently, C3 was detected with an anti-C3 fluorescein-conjugated goat IgG fraction detection antibody (Mpbio). Complement deposition is reported as mean fluorescent intensity (MFI) (fig. S3). Flow cytometry was performed with iQue (Intellicyt) and an S-Lab robot.

### Analysis of ADCP and ADNP

ADCP and ADNP were conducted according to the previously described protocols (*60*–*62*). In detail, SARS-CoV-2 antigens were biotinylated and coupled to yellow and green (505/515) fluorescent Neutravidin-conjugated beads (Thermo Fisher Scientific), respectively. To form immune complexes, antigen-coupled beads were incubated for 2 hours at 37°C with 1:100 diluted serum samples and then washed to remove unbound immunoglobulins. Following incubation, cells were fixed with 4% paraformaldehyde (Alfa Aesar). For ADCP, the immune complexes were incubated for 16 to 18 hours with THP-1 cells (American Type Culture Collection (ATCC)); 25,000 THP-1 cells per well at a concentration of 1.25×10^5^ cells/ml in R10 media (RPMI-1640 (Sigma-Aldrich) supplemented with 10% fetal bovine serum (FBS) (Sigma-Aldrich), 5% penicillin/streptomycin (50 μg/ml; Corning), 5% L-glutamine (4 mM; Corning), and 5% Hepes buffer (pH 7.2) (50 mM; Corning)) at 37°C, 5% CO2. The THP-1 based assay was optimized using good clinical laboratory practice (GCLP) standards, aiming to define the role of antibodies in shaping monocyte phagocytosis. However, the assay can be easily adapted to profile macrophage-like or dendritic cell-like activity through differential THP-1 maturation protocols. The protocol was established to maximize signal to noise for unmanipulated THP-1 activity. For ADNP, granulocytes were isolated from whole blood by lysing red blood cells (RBCs) in ACK lysis buffer (1:10 blood in ACK lysis buffer) for 7 min before precipitation by centrifugation. Granulocytes were washed twice with cold phosphate-buffered saline (PBS) and resuspended at 2.5×10^5^ cells/ml in R10 media; 50,000 cells per well were added to each well and incubated with immune complexes for 1 hour at 37°C, 5% CO_2_. For ADNP, RBC-lysed whole blood was washed, stained for CD66b^+^ (Pacific Blue–conjugated anti-CD66b clone G10F5 (2 μg/ml;) BioLegend, cat # 305112) to identify neutrophils, and then fixed in 4% PFA. Flow cytometry was performed to identify the percentage of cells that had phagocytosed beads as well as the number of beads that had been phagocytosed (phagocytosis score = % positive cells × Median Fluorescent Intensity of positive cells/10,000) (fig. S3). Flow cytometry was performed with 5 Laser LSR Fortessa Flow Cytometer, and analysis was performed using FlowJo V10.7.1.

### Antibody-dependent NK cell degranulation

Analysis of ADNKA was performed as described previously (*63*). Briefly, 96-well enzyme-linked immunosorbent assay (ELISA) plates were coated with indicated SARS-CoV-2 antigens at a protein concentration of 2 μg/ml (50 μl per well) and incubated at 37°C for 2 hours. After the incubation, plates were blocked with 5% bovine serum albumin (BSA) at 4°C overnight. NK cells were isolated from whole blood from healthy donors (by negative selection using RosetteSep (STEMCELL), according to the manufacturer's instruction) and then separated using a Ficoll gradient. NK cells were rested overnight in R10 media supplemented with interleukin (IL)-15 (1ng/ml of IL-15). The following day, serum samples (diluted 1:25) were added to coated plates, and immune complexes were allowed to form for two hours at 37°C. After the two hours, a cocktail of PE-Cy5 conjugated anti-CD107a antibody (clone H4A3; 2.5μl/well, 555802 BD Biosciences), brefeldin A (10 μg/ml, Sigma Aldrich), and GolgiStop (13.6μl of GolgiStop stock per 1ml of R10) was prepared in R10 medium (10 μl/well) and mixed with rested NK cells. NK cells were added to each immune complex-containing well at a concentration of 5x10^4^ NK cells per well (200μl/well) and incubated for 5 hours at 37°C. NK cells were surface stained with anti-CD3 Pacific Blue (clone UCHT1; 0.4 μl/well, BD Biosciences, cat # 558117), anti-CD16 allophycocyanin (APC)-Cy5 (clone 3G8; 1μl/well, BD Biosciences, cat # 557758), and anti-CD56 PE-Cy7 (clone B159; 1μl/well, BD Biosciences, cat # 557747) in PBS (total volume of antibody mix in PBS 10μl/well). Samples were incubated in the dark at room temperature for 15 min. Cells were washed twice with PBS, permeabilized (Perm A; Life Technologies), and washed twice with PBS again. MIP-1β-PE (clone D21-1351; 1μl/well, BD Biosciences, cat # 550078) was diluted in Perm B buffer (1:50 dilution) and added to each well (50μl). Samples were incubated in the dark for 15 min at room temperature. Cells were washed twice with PBS and resuspended in the final volume of 35μl PBS per well. Flow cytometry was performed with an iQue flow cytometer (Intellicyt). NK cells were gated as CD3^-^, CD16^+^, CD56^+^ cells, and NK cell activity was determined as the percent of NK cells positive for CD107a and MIP-1β (fig. S3).

### RBD-specific antibody depletion from polyclonal serum samples

D614G SARS-CoV-2 RBD-coated magnetic beads (ACROBiosystems) were prepared according to the manufacturer’s protocol and resuspended in ultrapure water at 1 mg/ml. Beads were washed three times in PBS with 0.05% BSA using a magnet. Serum samples were incubated with beads at 3:1 beads:serum ratio, rotating overnight at 4°C. A magnet was used to deplete beads with surface-bound RBD-specific antibodies. A mock depletion (pre-depletion samples) was performed by adding 150 μl of PBS and 0.05% BSA and incubating rotating overnight at 4°C. A standard ELISA was performed to confirm RBD-specific antibody depletion. Functional assays were performed with pre- and post-depletion samples.

### Statistics

Data analysis was performed using R version 4.0.2 (2020-06-22). All Luminex data were log-transformed, and all features were scaled. Comparisons between vaccination arms were performed using a Mann-Whitney U-test test followed by a Benjamini Hochberg (BH) correction. Antigen responses (such as D614G to Alpha) were compared using the Wilcoxon-signed rank test followed by BH correction. For RBD-specific antibody depletion, all data were Z-scored to visualize and compare differences in pre- and post-depletion functional results, and comparisons between samples were performed using paired *t* test.

Prior to any multivariate analysis, all data were normalized using Z-scoring. Multivariate classification models were trained to discriminate between individuals vaccinated with BNT162b2 and individuals vaccinated with mRNA-1273 using all the measured antibody responses. A PCA model was constructed using all antibody variables, including antibody titer, FcR measurements, and effector functions. Maximum separation was achieved in the two-dimensional space of PC1 (37.8%) versus PC2 (11.6%). A LASSO-PLSDA model was built using a combination of the least absolute shrinkage and selection operator (LASSO) for feature selection and then classification using partial least square discriminant analysis (PLS-DA) with the LASSO-selected features. Models were calculated and figures were generated using R package “ropls” version 1.20.0 (*64*) and “glmnet” version 4.0.2. Specifically, because antibody features are highly correlated (for example, IgG titers typically correlate with antibody effector functions), LASSO first captures the overall correlational structure of the data and identifies clusters of highly correlated features. LASSO then selects a single, or minimal, number of features from each cluster that best captures variation in that data group. The algorithm penalizes the selection of any additional features, aiming to use as few features as possible to define whether multivariate profiles differ across the groups. This reduced feature selection avoids statistical anomalies due to the over-representation of features that track together. Using this minimal set of features that best explains variation in the overall antibody profiles in the sample set, a final set of features is then used to determine whether groups exhibit similar or different profiles using PLSDA classification. LASSO was repeated 100 times, and features selected at least 90 times out of 100 were identified as selected features. A PLS-DA classifier was then applied to the training set using the selected features, and prediction accuracy was recorded. Model accuracy was then further assessed using ten-fold cross-validation. For each test fold, LASSO-based feature selection was performed on logistic regression using the training set for that fold. Selected features were ordered according to their Variable Importance in Projection (VIP) score, and the first two latent variables (LVs) of the PLS-DA model were used to visualize the samples. A co-correlate network analysis was carried out to identify features that highly correlate with the LASSO selected features, and thus are potentially equally important for discriminating the samples from individuals with each vaccination type. Correlations for the co-correlate network were performed using Spearman method followed by a BH correction for multiple comparisons (*65*). The co-correlate network was generated using R package “network” version 1.16.0 (*66*). All other figures were generated using ggplot2 (*67*).

## Supplementary Materials

### Other Supplementary Material for this manuscript includes the following:

MDAR Reproducibility Checklist
