# Supplementary material for: mRNA-1273 and BNT162b2 COVID-19 vaccines elicit antibodies with differences in Fc-mediated effector functions
Source: Sci Transl Med. 2022 Mar 29:eabm2311. doi: 10.1126/scitranslmed.abm2311 (PMC8995030; doi:10.1126/scitranslmed.abm2311)
Supplement: Supplementary file 1 — Fig. S1 to S3 [file scitranslmed.abm2311_sm.pdf]

Science Translational Medicine
AAAS

Supplementary Materials for

mRNA-1273 and BNT162b2 COVID-19 vaccines elicit antibodies with differences in Fc-mediated effector functions

Paulina Kaplonek *et al.*

Corresponding author: Galit Alter, alter@mgh.harvard.edu

DOI: 10.1126/scitranslmed.abm2311

The PDF file includes:

Figs. S1 to S3

Other Supplementary Material for this manuscript includes the following:

MDAR Reproducibility Checklist
Data files S1 to S3

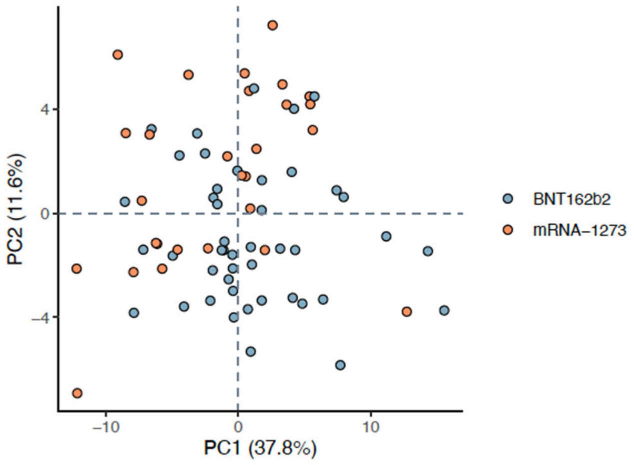

Fig. S1. An unsupervised principal component analysis (PCA) showed partial separation between BNT162b2 and mRNA-1273 vaccine profiles. An unsupervised PCA was performed on all antibody data generated across samples from BNT162b2 or mRNA-1273 vaccinees to examine whether differences exist in Fc-profiles induced by the different mRNA vaccines. PC1, principal component 1; PC2, principal component 2.

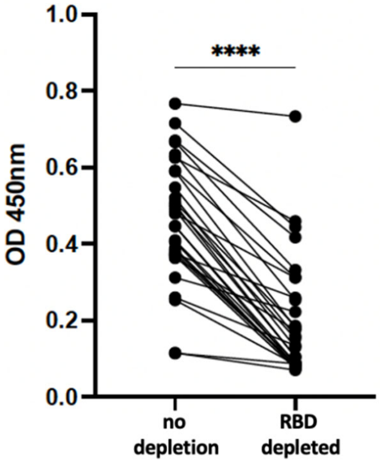

Fig. S2. Comparison of SARS-CoV-2 wild-type receptor binding domain (RBD)-specific antibody concentrations in serum samples before and after RBD-specific antibody depletion. SARS-CoV-2 RBD-specific antibody concentrations were determined by enzyme-linked immunosorbent assay in serum samples before (no depletion) and after RBD-specific antibody depletion (RBD depleted) at a 1:100 dilution. Statistical analysis was carried out with a paired parametric t-test (****$p < 0.0001$). OD, optical density.

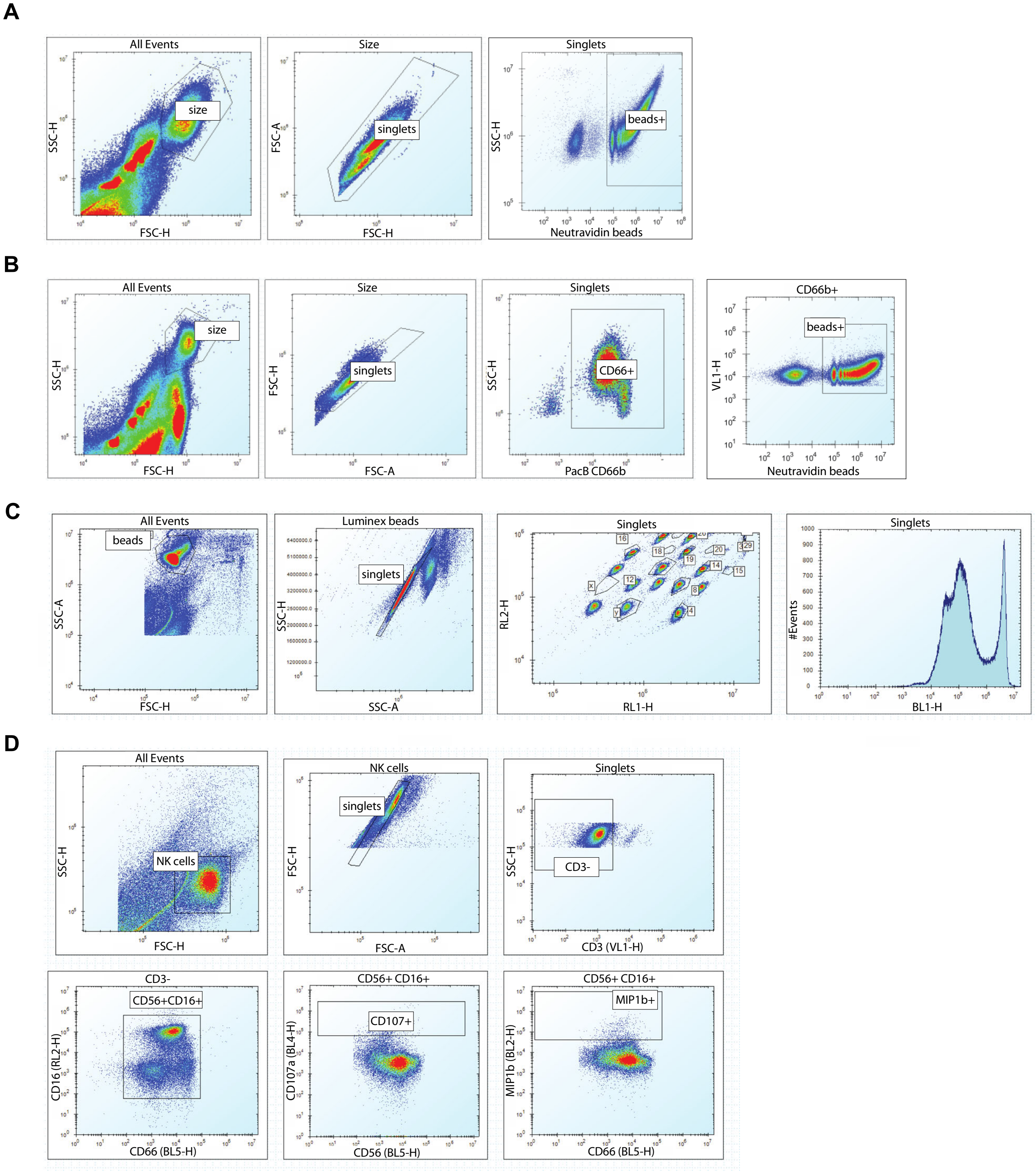

Fig. S3. Gating strategy for antibody-dependent effector assays. The diagram shows the flow cytometry gating strategy for **(A)** antibody-dependent cellular phagocytosis (ADCP) with THP-1 cells, **(B)** antibody-dependent neutrophil phagocytosis (ADNP) with primary human neutrophils, **(C)** antibody-dependent complement deposition (ADCD), and **(D)** antibody-dependent natural killer (NK) cell activation (ADNKA) with primary NK cells. SSC-H, side scatter height; FSC-H,

forward scatter height; FSC-A, forward scatter area; SSC-A, side scatter area; VL1-H, violet laser 1 height; RL1-H, red laser 1height; RL2-H, red laser 2 height; BL1-H, blue laser 1 height; BL5-H, blue laser 5 height ; BL6-H, blue laser 6 height.

Data file S1. Raw, individual-level data.

Data file S2. Statistical comparison of samples from BNT162b2 and mRNA1273 vaccine recipients.

Data file S3. Statistical comparison of samples from vaccine recipients and individuals with prior SARS-CoV-2 infection.
